# Association between dietary intake, energy availability, and recovery in young sub-elite Moroccan football players

**DOI:** 10.1080/15502783.2026.2730213

**Published:** 2026-09-09

**Authors:** El Mahdi Ait Aammi Hadi, El Mokhtar El Ouali, Anass Akhittouch, Hiba Rimy, Fadwa Zehhaf, Khalid Kassimi, Aziz Chokri, Abdeljalil Elgot, Mohamed Barkaoui

**Affiliations:** a Sports Science Research Team, Institute of Sports Sciences, Hassan 1st University, Settat, Morocco; b Laboratory of Health Sciences and Technologies, Higher Institute of Health Sciences, Hassan 1st University, Settat, Morocco

**Keywords:** Energy availability, energy intake, low energy availability, recovery, football

## Abstract

**Background:**

Energy availability is recognized as an important factor for health and performance in athletes, yet data in male football players and its relationship with post-exercise recovery remain limited. This study aimed to examine the associations between daily energy intake, energy availability, and recovery outcomes in football players.

**Methods:**

Nineteen outfield U21 players (*n* = 19) from a national championship in Morocco were monitored over four in-season weeks. On one training day, one rest day and one official match day per week, players completed 24-h dietary recalls, from which energy intake and macronutrient intake were quantified using Nutrilog software. Exercise energy expenditure was estimated and energy availability was calculated. Internal load was assessed using session rating of perceived exertion, subjective recovery with the Perceived Recovery Status scale 24-h post-session, and neuromuscular recovery via countermovement jump height.

**Results:**

Mean daily EI did not differ significantly between match, training, and rest days (*p* = 0.066), whereas EA was significantly lower on training days compared with match and rest days (both *p* ≤ 0.003). Higher EA was strongly associated with lower RPE (*ρ* = −0.597, *p* < 0.001) and modestly with higher PRS (*ρ* = 0.273, *p* = 0.001), while its association with CMJ was small and non-significant (*ρ* = 0.124, *p* = 0.132). Higher EI showed a moderate inverse correlation with RPE (*ρ* = −0.404, *p* < 0.001) and a small positive correlation with PRS (*ρ* = 0.173, *p* = 0.035), but no significant association with CMJ. Across EA categories, RPE, PRS and CMJ all differed significantly (*p* ≤ 0.014).

**Conclusion:**

Inadequate fueling relative to training demands leads to reduced EA, particularly on training days, and is associated with a lower recovery profile. Monitoring EA alongside simple field-based markers such as RPE and PRS may help practitioners identify periods of suboptimal fueling and adjust nutritional strategies accordingly.

## 1. Introduction

Football is considered as a high-intensity, intermittent sport that requires significant physical performance, quick recovery, and sustained energy levels from players to maintain a competitive advantage. Players must execute repeated high-intensity actions, such as sprints, accelerations, decelerations, and directional changes, that are alternating with periods of lower-intensity activity, thereby stimulating both aerobic and anaerobic energy systems [1,2]. Being able to quickly recover between these intense sessions is important for keeping up your performance during a match and during a busy competitive schedule [3].

Recovery in football is influenced by several strategies, including adequate sleep, nutrition, hydration, active recovery, stretching, massage, and cold-water immersion [4]. Nevertheless, football players may use an additional range of post-exercise recovery strategies than those above [5,6]. Sleep is one of the most important physiological restoration factors and also has an important impact on neuromuscular recovery, cognitive function and readiness for the next training session or competition. Inadequate sleep may negatively impact reaction time, decision-making, mood, perceived recovery, and physical performance. Recent evidence suggests that one night of sleep extension may improve physical and cognitive performance and highlights the importance of optimizing sleep as part of a multidimensional recovery approach in athletes [7].

Additionally, optimal nutritional strategies are essential in the recovery process given that they help restore energy reserves, support repair mechanisms, meet the high energy demands of football and maintain sufficient energy availability [8–10]. In this context, dietary intake and energy availability play a central role in supporting muscle regeneration, glycogen resynthesis and overall recovery processes [11].

Energy availability is defined as the dietary energy available for physiological functions after deducting the exercise energy expenditure, from total energy intake [10]. When energy availability is insufficient, the body prioritizes essential physiological functions, while other processes may receive inadequate energetic support [12].

Low energy availability (LEA) is a condition that occurs when the dietary energy intake is insufficient to cover the energy expenditure [13]. Athletes with LEA may experience disturbances in metabolism, hormonal balance, bone integrity, immune function, protein synthesis, hematologic function and cardiovascular efficiency, as well as growth and development. In female athletes, LEA can additionally disrupt menstrual function [13,14].

The existing literature on low energy availability (LEA) in male soccer players is significantly less comprehensive than that pertaining to females and endurance sports. To the best of our knowledge, only few studies have investigated energy availability in male football players to date [2,15,16]. Moreover, research on male soccer players has primarily focused on the prevalence and physiological correlations of low energy availability (e.g. metabolic suppression, endocrine alterations) without directly assessing its impact on post-exercise recovery outcomes. Therefore, this study aims to investigate the association between dietary intake, energy availability, and post-exercise recovery in male football players during an in-season competitive period.

## 2. Materials and methods

### 2.1. Ethical considerations

The Ethics Committee of the Mohammed VI University of Sciences and Health in Casablanca, Morocco, gave their approval for the study protocol [CE/UM6SS/22/25]. All procedures followed the Declaration of Helsinki. Before participating, each participant received verbal and written information on the goals and methodology of the study, and all signed a consent form to participate.

### 2.2. Study design and participants

A prospective observational study was carried out during four weeks in the competitive season when the under-21 squad were playing the national championship in Morocco. All players who regularly participated in team training and competitions were invited to participate. Twenty-three male outfield players (*n* = 23) volunteered to take part and to complete the monitoring phase. The inclusion criteria were: (i) being a player of the club's U21 team, (ii) not being injured at the start of data collection, (iii) attending all team training sessions and official matches during the study period, and (iv) being able to give informed consent . Players were excluded from analysis if they were injured for over 3 consecutive days, skipped more than two training sessions or failed to provide eating data for three or more days. Four participants were excluded from the study (*n* = 4), two due to injuries during the procedure, one due to non-attendance at the weekly anthropometric exam, and one due to incomplete food journals. Therefore, the final sample consisted of nineteen (*n* = 19) participants who finished the survey and were included in the analyses.

### 2.3. Data collection

**Figure 1.**
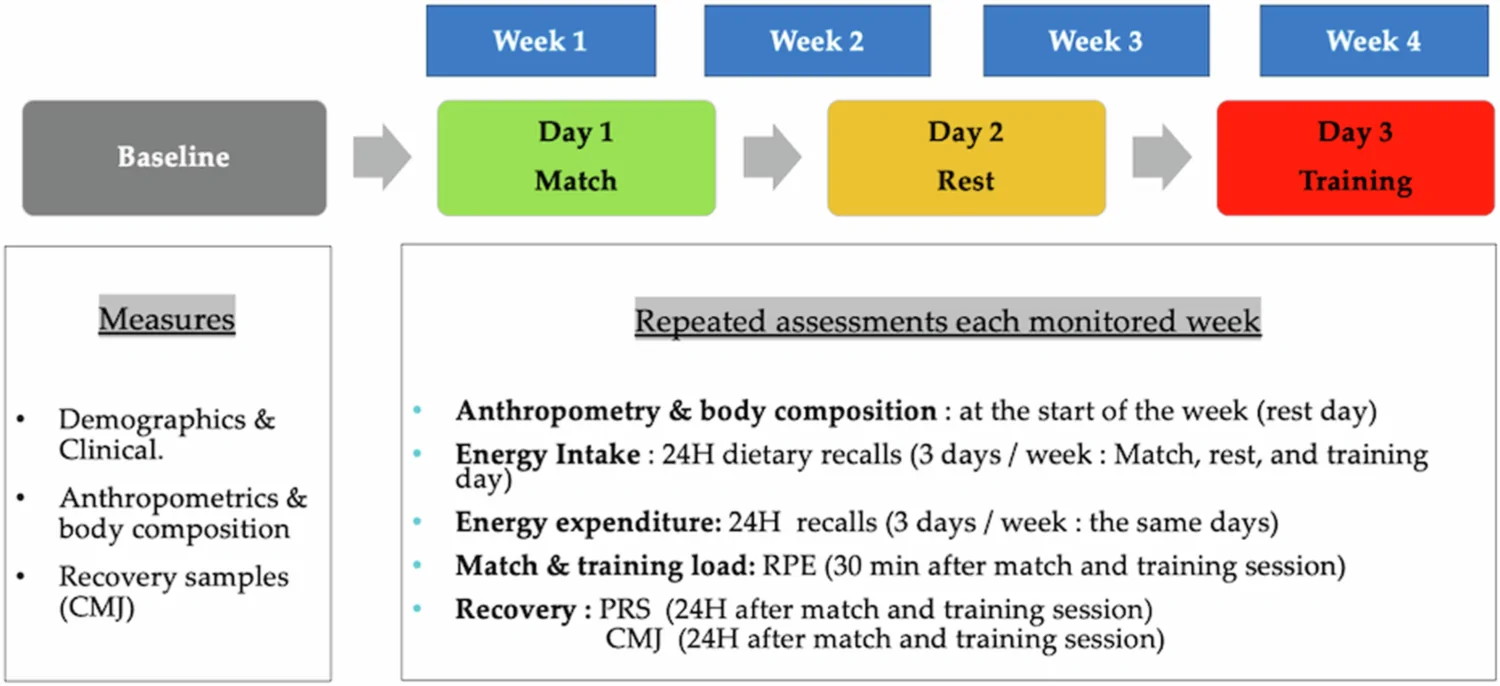
Flow diagram of the four-week study design and repeated assessments.

During a four-week period, data collection included one training session, one rest day, and one official match each week. We got demographic and clinical data at the start of the study. During the protocol time, we kept track of height, weight, body composition, food intake, energy expenditure, training and match load, and recovery data (Figure 1).

#### 2.3.1. Anthropometry and body composition

A wall-mounted stadiometer integrated with a medical scale (Seca 704s electronic scale with Seca 220 stadiometer, SECA GmbH, Hamburg, Germany), was used to measure height and body mass to the nearest 0.1 cm and 0.1 kg, respectively. Participants were barefoot and wore light clothing during the measurements. Body composition was assessed using a multi-frequency bioelectrical impedance analyzer (Bodystat1500, Bodystat Ltd., Douglas, Isle of Man, UK). Although dual-energy X-ray absorptiometry (DXA) is considered a more accurate method for assessing fat-free mass, bioelectrical impedance analysis (BIA) was used due to its field-based practicality and accessibility. To minimize variability which may be related to hydration status, measurements were performed, as far as feasible, under standardized conditions and participants were asked to avoid strenuous exercise, large meals, and excess fluid intake before the test. Resting metabolic rate (RMR) was calculated by the Harris–Benedict equation for males and expressed in kcal/day [17].

#### 2.3.2. Dietary intake

Dietary intake was assessed using 24-h dietary recalls for the monitored match, training, and rest days. To improve assessment accuracy and reduce recall bias, reported intakes were cross-checked using the club-provided menus for players residing at the training center. For players living outside the center, dietary intake was verified using continuous food diaries. In addition, structured interviews were conducted to clarify and correct information related to meal timing, food items, portion sizes, preparation methods, and possible omissions. All dietary assessments and verification procedures were conducted under the supervision of a nutritionist. A specialized nutrition analysis software (Nutrilog version 202509-23.1.7, Nutrilog SAS, Marans, France) was used to analyze food composition.

#### 2.3.3. Training and match load

Session-rating of perceived exertion (RPE) method was used to measure the internal training load. About 30 min after each training session and game, players used the [Borg 0–10] scale to rate how hard they thought they had worked, with 0 meaning “rest” and 10 meaning “maximal effort [18].” To lessen peer influence, RPE was gathered separately. The training load (arbitrary units, AU) was calculated as follows: Trainingload(AU)=Sessionduration(min)×RPE.

#### 2.3.4. Exercise energy expenditure

Exercise energy expenditure (EEE) was estimated using activity specific MET values from the compendium of physical activities [19]. This approach was used as continuous GPS, accelerometry, and heart rate monitoring were not available during the study period. Values were normalized with the official Compendium calculator [20]. Therefore, the data for EEE and EA values should be considered field based estimated values rather than direct measures.

#### 2.3.5. Energy availability

Energy availability (EA) was determined for each day of monitoring using the formula:

EA(kcal/kgFFM/day)=[EI(kcal)–estimatedEEE(kcal)]/FFM(kg)

[where EA is energy availability (kcal/kg FFM/day), EI is energy intake (kcal/day), EEE is exercise energy expenditure (kcal/day), and FFM is fat free mass (kg) [21].

The EA is divided into 3 categories: low EA (<30 kcal/kg FFM/day), moderate EA (30–45 kcal/kg FFM/day), and optimal EA (>45 kcal/kg FFM/day).

#### 2.3.6. Recovery assessment

##### 2.3.6.1. Subjective recovery (PRS)

Subjective recovery was assessed using the Perceived Recovery Status (PRS) scale [22]. Players rated their recovery on a scale of 0 to 10 (0 = “not recovered at all/extremely fatigued,” 10 = “fully recovered/ready to perform”) 24 h before any physical activity on training and match days. The study team monitored the gathering of individual PRS scores, which were recorded in a standard format.

##### 2.3.6.2. Neuromuscular recovery (countermovement jump)

The recovery of the neuromuscular system was measured using the countermovement jump (CMJ) test. Testing was administered in the morning of the monitoring days prior to the first training session and following a predetermined warm-up. Participants performed countermovement jumps with their hands on their hips to restrict arm movement. They started from a standing position, immediately fell to a depth of their choosing, then jumped straight up “as fast and as high as possible.” A Chronojump BoscoSystem, Barcelona, Spain was used to assess jump height. After 1–2 familiarization trials, participants completed 2 maximal countermovement jumps with a minimum rest interval of 30 s between attempts. The maximum jump height was kept for examination and used as an objective indicator of neuromuscular recovery.

#### 2.3.7. Statistical analysis

Descriptive statistics are presented as mean ± standard deviation and 95% confidence intervals. The normality assumption for all continuous variables was assessed using the Shapiro–Wilk test. The initial analysis plan considered parametric options for data that follows a normal distribution. Because of large deviations from normality and the presence of outliers in several key variables and residuals, non-parametric tests were considered appropriate and consequently employed for all inferential studies. Thus, correlations between EI, EA, and recovery outcomes (RPE, PRS, and CMJ) were analyzed using Spearman's rank correlation coefficients (*ρ*), which furthermore functioned as impact size indices.

The Friedman test, a non-parametric alternative to repeated-measures ANOVA, was employed to analyze variations in EI and EA over match, training, and rest days, with Kendall's W serving as the effect size measure. Upon detecting a significant main effect, pairwise comparisons were performed using Wilcoxon signed-rank tests with Bonferroni-adjusted significance levels. To examine differences in recovery indices among categories of EA, individual days were classified as low, moderate, or optimal EA and analyzed using the Kruskal–Wallis test (the non-parametric equivalent of one-way ANOVA), followed by Mann–Whitney U tests with Bonferroni correction for subsequent pairwise comparisons. Effect size r for the Wilcoxon and Mann–Whitney tests was calculated using the formula r =| *Z*|√*N*, where *Z* represents the standardized test statistic and *N* denotes the number of observations.

The post hoc sensitivity power analysis was performed using G*Power software version 3.1.9.6. The final sample size of 19 players was sufficient to detect large associations of r ≥ 0.59 (two-tailed test of correlation; alpha level of 0.05; statistical power of 80%). Thus, the sample size was considered sufficient to detect large effects, however, the study was probably underpowered to detect small to moderate associations [23].

The threshold for statistical significance was set at *p* < 0.05. All statistical analyses were performed using IBM SPSS Statistics, version 26 (IBM Corp., Armonk, NY, USA).

## 3. Results

The players' descriptive characteristics, mean daily nutritional intake, energy expenditure, and the normality of all variables are presented in Table 1. The primary inferential analyses examining energy intake (EI), energy availability (EA), and recovery outcomes (RPE, PRS, and CMJ) are summarized in Tables 2–4.

**Table 1.** Descriptive characteristics, body composition, energy expenditure, dietary intake, energy availability, and recovery indices in U21 male football players (*n* = 19).

Variables	Descriptive statistics			Normality	
	Range	Mean	SD	Shapiro–Wilk	*p*-value
Age (years)	(18; 21)	18.9	0.9	0.831	0.003
Height (cm)	(161; 190)	177.2	6.5	0.965	0.669^a^
Body mass (kg)	(57.9; 86.1)	70.5	7.8	0.936	0.001
BMI	(19.30; 27.48)	22.45	2.29	0.933	0.001
Body fat (%)	(8.10; 17.90)	12.72	2.42	0.943	0.002
FFM (Kg)	(50.40; 71.60)	60.84	5.89	0.952	0.006
BMR (Kcal)	(1539.88; 2015.74)	1794.64	123.16	0.980	0.937^a^
EEE (Kcal·day^−1^)	(0.00; 2355.70)	774.39	615.37	0.932	<0.001
Absolute EI (Kcal·day^−1^)	(218; 3205)	1831.60	608.89	0.990	0.126^a^
EA (Kcal kg^−1^·day^−1^)	(−14.97; 55.37)	17.18	14.51	0.979	0.002
Relative EI (Kcal·kg^−1^)	(2.67; 44.67)	26.18	8.58	0.982	0.005
Total carbohydrates (g)	(28.30; 463.00)	215.54	97.74	0.972	0.017
Relative carbs(g·kg^−1^)	(0.30; 6.70)	3.06	1.40	0.966	0.005
Total protein (g)	(11.50; 193.10)	91.49	44.99	0.963	0.003
Relative protein (g·kg^−1^)	(0.10; 2.80)	1.31	0.64	0.967	0.006
Total fat (g)	(0.00; 147.90)	58.82	33.58	0.964	0.003
Relative fat (g·kg^−1^)	(0.00; 2.20)	0.84	0.47	0.964	0.004
RPE (AU)	(0; 900)	552.56	310.91	0.845	<0.001
PRS	(5; 9)	6.66	1.28	0.783	<0.001
CMJ (cm)	(20.69; 38.26)	29.43	3.86	0.971	0.003

Values are presented as range. mean, and standard deviation (SD).

^a^ Normality was assessed using the Shapiro–Wilk test; variables with *p* ≥ 0.05 were considered normally distributed.

**Table 2.** Correlations between energy availability, energy intake, and recovery indices in U21 male football players (*n* = 19).

Variables	Test (statistic, df)	*p*-value	Effect size^b^
EA ⇔ RPE	*ρ* = −0.597	<0.001^a^	*ρ* = −0.597 (large)
EA ⇔ PRS	*ρ* = 0.273	0.001^a^	*ρ* = 0.273 (small)
EA ⇔ CMJ	*ρ* = 0.124	0.132	*ρ* = 0.124 (small)
EI ⇔ RPE	*ρ* = −0.404	<0.001^a^	*ρ* = −0.404 (moderate)
EI ⇔ PRS	*ρ* = 0.173	0.035^a^	*ρ* = 0.173 (small)
EI ⇔ CMJ	*ρ* = 0.133	0.104	*ρ* = 0.133 (small)

^a^ Values are Spearman's rank correlation coefficients (ρ) with corresponding *p*-values. Statistically significant correlations (*p* < 0.05).

^b^ Effect sizes are interpreted as small (|ρ| ≈ 0.10), moderate (|ρ| ≈ 0.30), and large (|ρ| ≥ 0.50).

**Table 3.** Comparisons of energy intake and energy availability across match, training, and rest days in U21 male football players (*n* = 19).

Variables	Test (statistic, df)	*p*-value	Effect size^c^
EI across match, training, and rest days	Friedman χ^2^(2) = 5.44	0.066	Kendall's W = 0.14 (small)
EA across match, training, and rest days	Friedman χ^2^(2) = 11.79	0.003^a^	Kendall's W = 0.31 (moderate)
EA match vs rest	Wilcoxon Z = −0.885	0.376	r = 0.20 (small)
EA training vs match	Wilcoxon Z = −2.94	0.003^b^	r = 0.67 (large)
EA training vs rest	Wilcoxon Z = −3.30	0.001^b^	r = 0.76 (large)

^a^ Significant for Friedman tests (overall comparison across day types) at *p* < 0.05.

^b^ Wilcoxon signed-rank tests (pairwise post-hoc comparisons), with corresponding significant *p*-values < 0.017.

^c^ Kendall's W is reported as an effect size for Friedman tests (≈0.10 small, ≈0.30 moderate, ≥0.50 large). Effect size r for Wilcoxon tests was derived from the Z statistic (r = |Z|/√N), where *N* represents the number of paired observations (19 players), and was interpreted as small (≈0.10), moderate (≈0.30), or large (≥0.50).

**Table 4.** Comparisons of recovery indices across energy availability categories in U21 male football players (*n* = 19).

Variables	Test (statistic, df)	*p*-value	Effect size^c^
RPE vs EA categories	Kruskal–Wallis H(2) = 59.45	<0.001^a^	η^2^_H = 0.38 (large)
PRS vs EA categories	Kruskal–Wallis H(2) = 14.67	0.001^a^	η^2^_H = 0.09 (moderate)
CMJ vs EA categories	Kruskal–Wallis H(2) = 8.58	0.014^a^	η^2^_H = 0.04 (small)
RPElow vs moderate EA	Mann–Whitney Z = −7.37	<0.001^b^	r = 0.60 (large)
PRS low vs moderate EA	Mann–Whitney Z = −3.76	<0.001^b^	r = 0.31 (moderate)
CMJ low vs moderate EA	Mann–Whitney Z = −2.45	0.014^b^	r = 0.20 (small)
RPE low vs optimal EA	Mann–Whitney Z = −2.79	0.005^b^	r = 0.25 (small)
PRS low vs optimal EA	Mann–Whitney Z = −0.60	0.548	r = 0.05 (very small)
CMJ low vs optimal EA	Mann–Whitney Z = −1.72	0.085	r = 0.16 (small)
RPE moderate vs optimal EA	Mann–Whitney Z = −0.39	0.699	r = 0.07 (very small)
PRS moderate vs optimal EA	Mann–Whitney Z = −1.44	0.149	r = 0.27 (small)
CMJ moderate vs optimal EA	Mann–Whitney Z = −1.08	0.281	r = 0.20 (small)

^a^ Significant for Kruskal–Wallis tests (overall comparison across EA categories (low, moderate, and optimal)) at *p* < 0.05.

^b^ Mann–Whitney U tests (post-hoc pairwise comparisons), with corresponding *p*-values < 0.017.

^c^ For Kruskal–Wallis tests, η²_H was calculated as (H − (*k* − 1))/(*N* − 1), where *k* is the number of groups and *N* the total number of observations, and interpreted as small (~0.01), moderate (~0.06) or large (≥0.14). For Mann–Whitney tests, effect size r was derived from the Z statistic (r = |*Z*|/√*N*) and interpreted as very small (<0.10), small (~0.10), moderate (~0.30), or large (≥0.50).

Spearman's rank correlation analyzes indicated that elevated daily EA was significantly associated with lower perceived exertion (RPE; *ρ* = −0.597, *p* < 0.001) and higher perceived recovery at 24 h (PRS; *ρ* = 0.273, *p* = 0.001), although the association with CMJ performance at 24 h was small and not statistically significant (*ρ* = 0.124, *p* = 0.132). Similarly, higher EI was moderately negatively correlated with RPE (*ρ* = −0.404, *p* < 0.001) and slightly positively correlated with PRS (*ρ* = 0.173, *p* = 0.035), but not significantly correlated with CMJ (*ρ* = 0.133, *p* = 0.104) (Table 2).

However, no significant difference was observed in energy intake between match, training, and rest days (Friedman χ2(2) = 5.44, *p* = .066, Kendall's W = 0.14, small effect) suggesting that players generally maintained a similar energy intake across day types. Conversely, EA exhibited significant variation across day types (Friedman χ^2^(2) = 11.79, *p* = 0.003, Kendall's W = 0.31, moderate effect). Post-hoc Wilcoxon tests indicated that EA was significantly lower on training days than on match days (Z = −2.94, *p* = 0.003, r = 0.67, large effect) and rest days (Z = −3.30, *p* = 0.001, r = 0.76, large effect), while no significant difference was observed between match and rest days (Z = −0.885, *p* = 0.376, r = 0.20, small effect) (Table 3).

**Figure 2.**
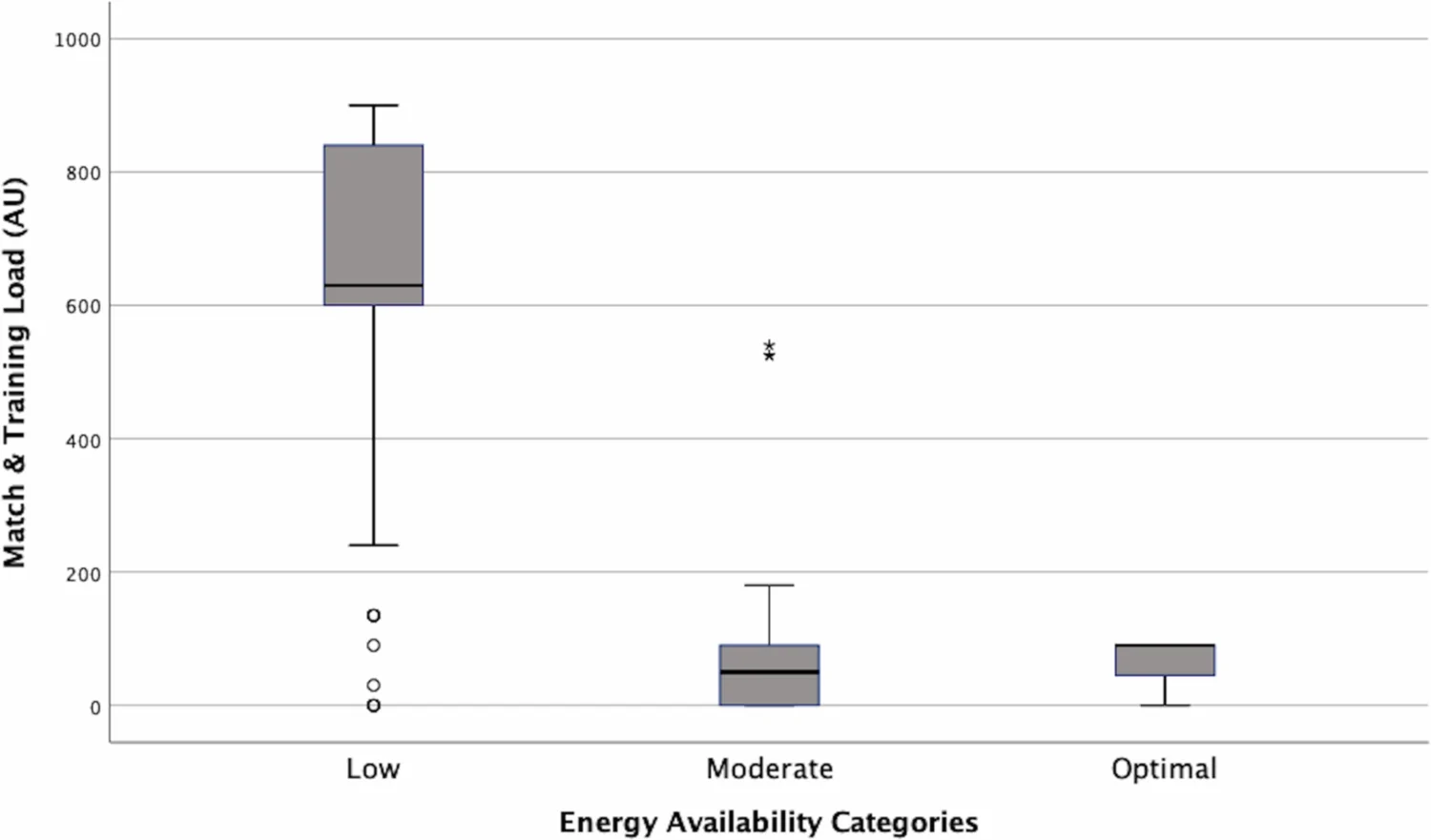
Session rating of perceived exertion across energy availability categories.

**Figure 3.**
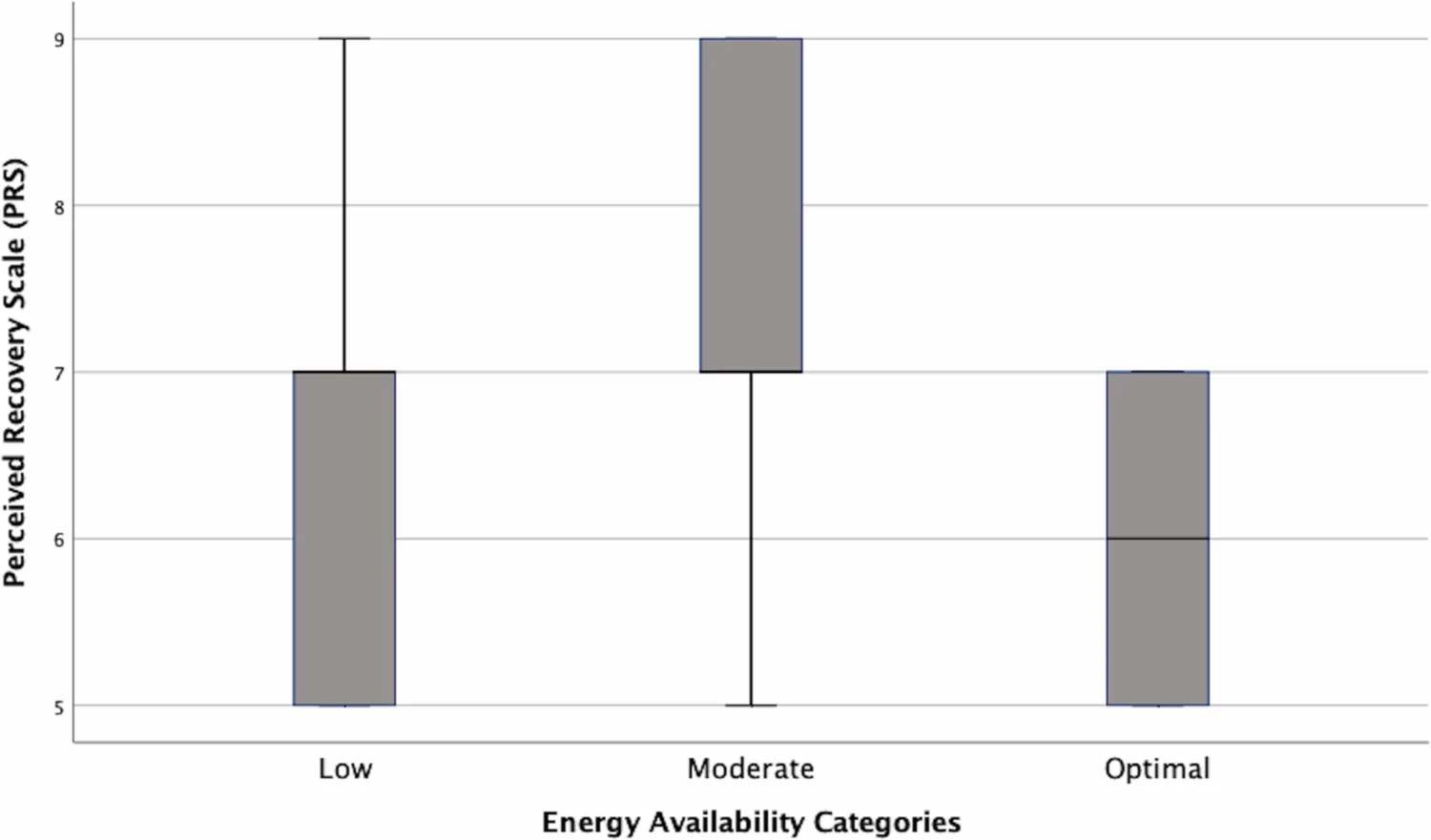
Perceived recovery scale across energy availability categories.

**Figure 4.**
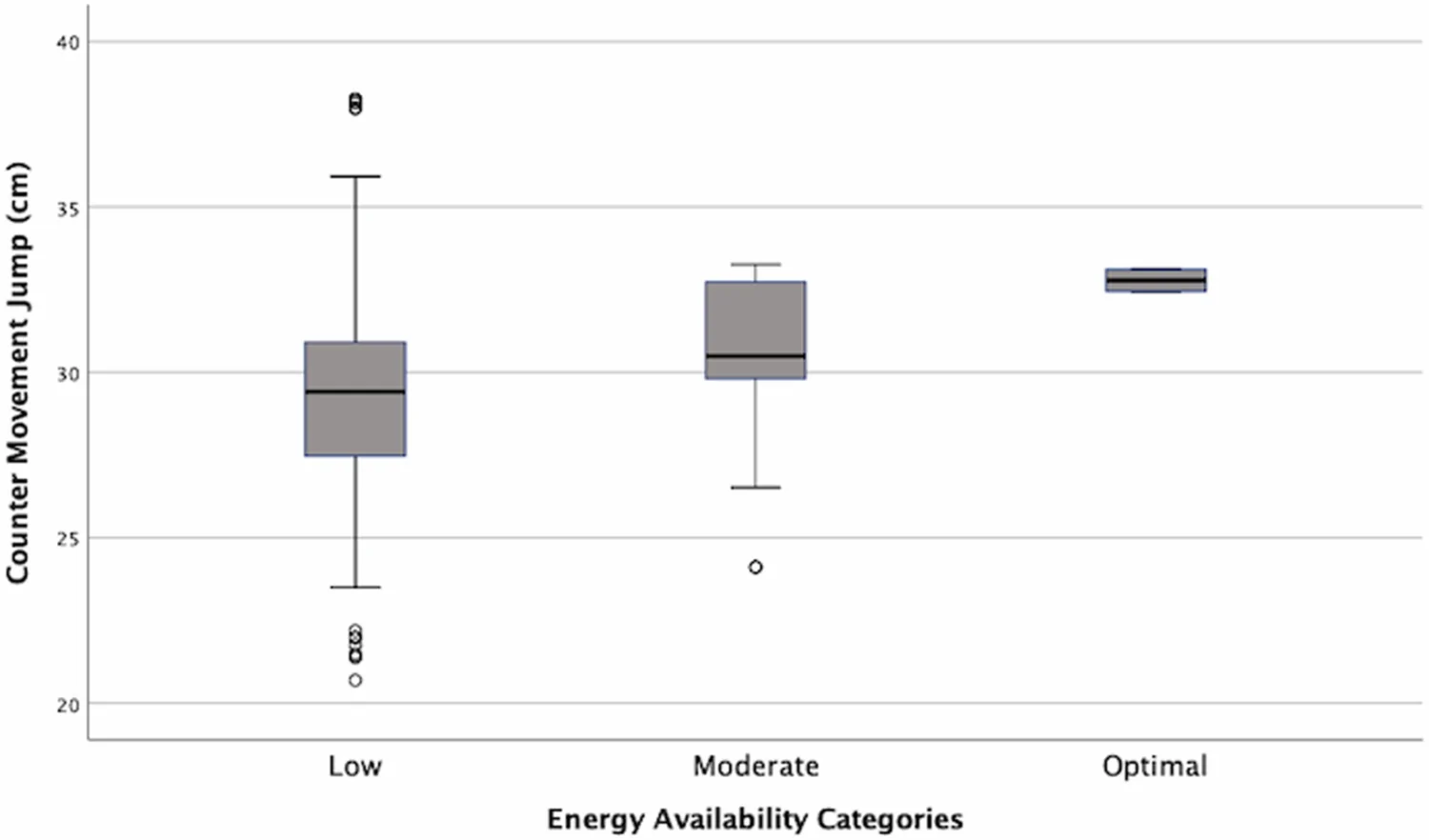
Countermovement jump across energy availability categories.

Following the classification of days into low, moderate, and optimal EA, the Kruskal–Wallis test revealed significant intergroup differences across all three recovery indices: RPE (H(2) = 59.45, *p* < 0.001) (Figure 2), PRS (H(2) = 14.67, *p* = 0.001) (Figure 3), and CMJ (H(2) = 8.58, *p* = 0.014) (Figure 4). Post-hoc Mann–Whitney tests revealed that, in comparison to moderate EA, low EA days exhibited higher RPE (Z = −7.37, *p* < 0.001, r = 0.60, large effect), lower PRS (Z = −3.76, *p* < 0.001, r = 0.31, moderate effect), and lower CMJ performance (Z = −2.45, *p* = 0.014, r = 0.20, small effect). Compared with optimal EA, low EA days were associated with higher RPE (Z = −2.79, *p* = 0.005, r = 0.25, small effect), whereas differences in PRS (Z = −0.60, *p* = 0.548, r = 0.05, very small effect) and CMJ (Z = −1.72, *p* = 0.085, r = 0.16, small effect) did not reach statistical significance. No significant differences were observed between moderate and optimal EA for RPE (Z = −0.39, *p* = 0.699, r = 0.07, very small effect), PRS at 24 h (Z = −1.44, *p* = 0.149, r = 0.27, small effect), or CMJ at 24 h (Z = −1.08, *p* = 0.281, r = 0.20, small effect) (Table 4).

## 4. Discussion

This study aimed to investigate the associations between daily energy intake, energy availability, and recovery in U21 male football players during a 4-week in-season period using an observational prospective method. The main findings indicate that the average daily energy intake did not show significant differences between match, training, and rest days; nevertheless, energy availability significantly decreased on training days in comparison to both match and rest days. Upon further classification of daily observations into energy availability categories (low, moderate, and optimal), significant variations were observed in the recovery indices, with low energy availability days characterized by higher perceived exertion, poorer subjective recovery and slightly reduced CMJ height. Few studies have particularly investigated the effect of LEA on post-exercise recovery parameters. Only two studies have indicated that athletes with LEA report a signifi cant feeling of under-recovery [24,25]. The majority of existing research have focused on performance outcomes or health-related effects among athletes, especially among female individuals.

### 4.1. Energy intake and carbohydrates distribution

Daily dietary intake did not show significant differences between match, training, and rest days (1946.41, 1928.80, and 1651.99 kcal, respectively). Typically, nutritional intake peaks on match days, be moderate on training days, and lowest on rest days [2,9,26], reflecting the different physical requirements of each type of day. These findings are partially consistent with this pattern, since rest days generally presented the lowest intake; however, we did not detect a statistically significant increase in energy intake on match days compared to training days. A similar absence of distinct daily variations has been observed in certain youth and female teams, where dietary practices are less controlled and athletes typically adhere to a rather stable diet throughout the week, irrespective of training intensity [27,28]. These patterns are observed across several levels of competition, including professional, youth, and female players, and are reflected in associated team sports [29–31]. The data indicate that several footballers, including those in the present study, may not adequately periodize their energy intake to correspond with variations in external load, potentially leading to periods of LEA and inadequate recovery. Furthermore, the relative contribution of macronutrients, particularly carbohydrates, to total EI appears to be insufficient in this group. On average, players ingested approximately 3 g/kg/day of carbohydrates during the monitored days, which is significantly below the current guidelines for team-sport athletes, typically ranging from 3 to 8 g/kg/day during in-season training with one match weekly [8], and from 6 to 10 g/kg/day during congested fixture periods [3,8]. This indicates that, even though daily EI was not dramatically low, the carbohydrate contribution to total energy was insufficient to support repeated high-intensity efforts and glycogen resynthesis between sessions [32]. Conversely, studies that incorporated nutrition education or individualized fueling strategies generally demonstrate increased carbohydrate availability and improved adherence to recommendations, especially during matches [33,34]. Consequently, the findings in this research, contribute to the emerging evidence that, the insufficient carbohydrate availability likely contributed to the lower EA reported on training days and may partially explain the less favorable recovery observed on days classified as having LEA [35].

### 4.2. Energy availability across day types

EEE was estimated using activity specific MET values from the Compendium of Physical Activities. Therefore, the EEE and EA values reported in the present study should be considered as estimates and may be subject to measurement error. Furthermore, the threshold of low energy availability (EA) is often categorized as EA < 30 kcal/kg FFM/day, however, this threshold was mainly determined in female athletes and its direct application to male team sport athletes remains uncertain. Therefore, the EA categories in this study should be seen as indicative rather than diagnostic. Accordingly, EA showed a distinct pattern across day types. It was markedly reduced on training days (9.32 kcal/kg FFM/day) compared with both match days (19.57 kcal/kg FFM/day) and rest days (22.99 kcal/kg FFM/day). Previous research has also shown that EA is often lowest on match and training days (i.e. days with the greatest exercise energy expenditure), and increases on rest days [36,37], a pattern that has been attributed to inadequate nutritional periodization, particularly suboptimal carbohydrate intake. Although, some studies in professional teams have shown the lowest EA on match days due to the partial compensation of training loads through tactical rotation and decreased training volume [26]. The results suggest that, in this U21 cohort, players did not sufficiently adjust their food intake to compensate for the higher exercise energy expenditure on training days, resulting in a recurrent decline in EA on these days. LEA may lead to a range of detrimental health and performance consequences for football players [38]. The proportion of days falling below this threshold in the present study suggests that LEA may frequently occur in academy football, despite the absence of evident weight loss or clinical signs.

### 4.3. Recovery indices across energy availability categories

Recovery was assessed using PRS and CMJ, practical and commonly used field-based measures. However, these indicators may not represent the multifactorial nature of recovery in its entirety. Future research should combine PRS and CMJ with objective indicators as HRV, RHR, sleep monitoring, biochemical markers or validated recovery-monitoring devices. Ardigò et al. reported the external responsiveness of the SuperOp™ device for the assessment of recovery after exercise, supporting the value of device-based tools as complementary methods for recovery monitoring in athletes [39]. Nevertheless, the primary contribution of this work is the combination of EA with other recovery indicators. Increased daily energy availability correlated with reduced session RPE and elevated PRS, however no definitive relationship was observed with CMJ performance. On days with poor energy availability (EA), there was a notable increase in felt exertion, a decrease in perceived recovery, and a modest reduction in CMJ compared to days with moderate or optimal EA. The inverse association between EA and RPE should be interpreted with caution due to the potential circularity in the calculation of EA. Therefore, this relationship may in part be due to the fact of calculation method rather than an independent physiological association. These results are in line with previous research indicating that athletes with LEA are more vulnerable to experience fatigue, sleep issues, and under-recovery [24,25]. Nonetheless, not all research has identified robust correlations between EA and short-term neuromuscular indicators such as countermovement jump or sprint performance; in many populations, performance declines manifest only after extended durations of LEA or in reaction to intensive training phases [40,41]. The findings of the current research corroborate this distinction: subjective markers (RPE, PRS) shown greater sensitivity to daily variations in EA compared to CMJ, indicating that internal load and perceived recovery may serve as earlier indicators of inadequate fueling than basic field performance measurements.

### 4.4. Performance and health implications of LEA

The considerable effects on health of LEA are especially pertinent in young athletes. Compared to athletes with optimal EA, athletes with LEA have been shown to demonstrate impaired running performance, reduced training adaptation, decreased agility and decrements in aerobic and anaerobic performance markers such as VO₂max, peak power output, relative power output and anaerobic threshold [42,43]. They also report greater fatigue and sleep disturbances [25], more frequent absence from training owing to illness [44], and overall lower performance and delayed recovery [25]. In youth soccer players, chronic LEA may further compromise growth and maturation, impair bone mineral accrual, delay sexual maturation, and alter immune function [15]. The findings of the present study indicate recurring LEA days and an adverse recovery profile in U21 athletes, supporting these concerns and suggesting that inadequate fueling may already manifest at an early stage when health and performance trajectories are still being defined.

Finally, several studies have suggested that athletes with LEA appear to be at increased risk of sports injuries [25,45], particularly, bone stress injuries [43,46,47]. Additionally, LEA may also contribute to an increased risk of sports injury by altering fatigue, neuromuscular function, tissue repair, and movement quality. Energy deficits may reduce muscle strength and power generation, impair coordination, and alter biomechanical patterns during high-intensity activities including sprinting, cutting, and landing. In football, these changes may impair the athlete's ability to tolerate repeated mechanical loads, and may increase the risk of injury. Therefore, for players at risk of LEA, injury prevention requires a multidimensional tracking approach that includes nutritional status, training load, neuromuscular performance, recovery markers and biomechanical assessment [48]. This study did not evaluate injury outcomes and thus cannot directly answer this question; however, the significant prevalence of LEA days highlights the necessity for longitudinal research connecting objectively monitored energy availability, recovery responses, and patterns of injury and illness in youth football players.

### 4.5. Practical implications

These findings underscore the necessity for systematic nutritional periodization in U21 football players, particularly on training days, which were identified as the most susceptible to LEA. Practical nutritional periodization needs to take into account not only adequate total energy intake but also specific nutrient timing in relation to training, competition, and sleep. Timing of protein intake may aid recovery and subsequent performance in soccer players. Bayrakdaroğlu et al. reported that both post-exercise and pre-sleep casein ingestion improved anaerobic performance in highly trained soccer players without any clear advantage of one strategy over the other. Thus, protein intake after exercise and/or before sleep could be flexible strategies for recovery in individualized nutrition plans [49]. Furthermore, coaches and support personnel should ensure that daily energy and, particularly, carbohydrate consumption is adjusted according to session requirements, aiming to meet the current guidelines for team-sport athletes during intensive training and competition days. Inexpensive, simple methods like session RPE and PRS can be incorporated into standard monitoring to identify days or weeks when athletes report significantly elevated perceived exertion or inadequate recovery, necessitating an evaluation of nutritional intake and training load. Educational initiatives for academy players and their families regarding fueling techniques, pre- and post-training snacks, and carbohydrate scheduling could reduce the occurrence of low energy availability days and enhance both performance and long-term health.

### 4.6. Limitations

To the authors' knowledge, this is the first study examining the association between dietary intake, energy availability and recovery in young male football players. However, there are several limitations to consider. The primary limitation of the current study is the use of Compendium derived MET values to estimate exercise energy expenditure rather than direct or wearable based methods such as GPS, accelerometry, heart-rate monitoring or doubly labelled water. This limitation is particularly relevant in football where the stochastic and intermittent nature of the demands during a match and training imply frequent changes in speed, accelerations, decelerations, high-intensity running, technical actions, and rest periods. Therefore, MET-based estimates may not capture individual differences in external and internal load, playing position, fitness level, and physiological responses. Since EEE is directly involved in the calculation of EA, any error in estimation of EEE could have influenced the accuracy of the EA values and the classification of players into EA categories. Thus, the EA data presented in this study should be considered as approximate field-based estimates, with potential error margins, rather than exact measurements.

Second, the use of BIA rather than DXA to estimate FFM is an important limitation, as changes in hydration may affect BIA-derived FFM and, consequently, EA calculation and classification. Although body composition assessments were performed in standardized field conditions, future studies should employ DXA, ISAK standards or other reference methods to improve precision of FFM assessment.

Third, for players living in the training center, dietary intake was validated by club menus, while for players living outside of the training center, dietary intake was validated by continuous dietary records. However, the use of 24-h dietary recalls can still be subject to recall bias and day-to-day variability. Future studies should consider the use of weighed food records, photographic food records or digital dietary tracking tools to further improve the accuracy of dietary assessment.

Fourth, the sample size was limited and taken from one U21 male team from one club, limiting the generalizability of the findings to other age groups, competitive levels, clubs, and female athletes. In addition, the observational period was restricted to four in-season weeks with one match per week, so different energy availability and recovery patterns may take place during pre-season periods, congested fixture schedules, or other periods of the competitive season.

Fifth, recovery was determined by PRS and CMJ with no other objective autonomic or physiological markers such as HRV, RHR, sleep monitoring, biochemical markers, hormonal markers, or bone health indicators. PRS was measured at 24 h post-session without adjustment for individual baseline PRS scores or external stressors such as sleep quality, psychological stress, muscle soreness, academic or personal demands, and previous training load. This may have limited the validity of PRS as a standalone recovery indicator. Finally, long-term injury, illness, or health outcomes were not assessed in this study. Consequently, the potential health effects of recurrent days of low energy availability in this population remain poorly defined.

## 5. Conclusion

The present study shows that young male competitive football players commonly experience several days of low energy availability during the in-season period, primarily due to insufficient adjustment of energy intake, particularly carbohydrate intake, to meet training demands. Total daily energy intake did not differ significantly between match, training, and rest days, however, estimated energy availability was significantly lower on training days. Overall, low energy availability days were associated with higher perceived exertion, poorer subjective recovery, and slightly lower countermovement jump performance. These findings indicate that insufficient fueling may initially manifest as a greater internal load and compromised perceived recovery before overt reductions in physical performance are observed. However, due to the estimation of exercise energy expenditure in the field, the findings should be interpreted with caution and the reported energy availability values should be considered as approximate estimates, rather than exact measurements. Together, these findings highlight the need for regular assessment of energy availability in conjunction with field-based recovery markers and targeted nutritional approaches (e.g. carbohydrate periodization and personalized energy intake planning) to promote training adaptation, recovery and long-term health in U21 football players.
